# Predictive value of immediate pain relief after lumbar transforaminal epidural injection with local anesthetics and steroids for single level radiculopathy

**DOI:** 10.1007/s00256-022-04051-3

**Published:** 2022-04-08

**Authors:** Christoph Germann, Tobias Götschi, Reto Sutter

**Affiliations:** 1 Radiology, Balgrist University Hospital, University of Zurich, Forchstrasse 340, CH-8008 Zurich, Switzerland; 2 Balgrist Campus AG, Lengghalde 5, 8008 Zurich, Switzerland

**Keywords:** Radiculopathy, Injections, Steroids, Anesthetics, Treatment outcome

## Abstract

**Objective:**

To assess the predictive value of immediate pain-relief after CT-guided transforaminal epidural steroid injection (TFESI) including local anesthetics for longer-term pain relief and patients’ global impression of change (PGIC) after 4 weeks.

**Materials and methods:**

One hundred ninety-three patients (age 55.4 ± 14.9) with single-level discogenic lumbar radiculopathy and subsequent TFESI were included. Pain scores were recorded before (NRS_0_), 15 min (NRS_15min_), and 4 weeks (NRS_4w_) after treatment using a numerical-rating-scale (NRS; 0, no pain; 10, intolerable pain). Additionally, the PGIC was assessed after 4 weeks. Two fellowship-trained musculoskeletal radiologists evaluated nerve compression of the injected level and contrast dispersion. Spearman’s rank and point-biserial correlation were applied to assess associations between outcome variables and demographics/imaging findings. A *p*-value < 0.05 was considered to be statistically significant.

**Results:**

There was a significant positive correlation between immediate pain-relief and longer-term pain-reduction (*r* = 0.24, *p* = 0.001) with an odds ratio of 2.0 (*CI*: 1.1–3.6). A good short-term response (NRS_15min_ ≥ 50% reduction) was associated with a persistent longer-term good response (NRS_4w_ ≥ 50% reduction) in 59.7% (*CI*: 50.9–68.0%) of patients. There was no association between short-term pain-relief and PGIC after 4 weeks (*p* = 0.18). Extent and location of nerve compression and contrast dispersion during TFESI did not correlate with longer-term pain-relief (all *p* ≥ 0.07).

**Conclusion:**

Our results indicate a significant positive correlation between immediate post-procedural and longer-term pain relief after TFESI in patients with lumbar radiculopathy; however, no effect of short-term pain relief is seen on PGIC after 4 weeks. Patients with good longer-term outcome (≥ 50% pain reduction) are twice as likely to have already shown good immediate pain reduction after TFESI.

## Introduction

Lumbar radiculopathy as part of the spectrum of low back pain is a frequent symptom seen in patients in primary care clinics and is a common cause of activity limitation and work absence, posing a major socioeconomic concern [1–3]. Various pathologic conditions or any combination of those may cause lumbar radiculopathy. Among the most common are disc herniation, spondylosis, osteoarthritis of facet joints, and hypertrophy of the ligamentum flavum. Each of these conditions may lead to a mechanical compression of nerves, either within the spinal canal, at the lateral recess, within the neuroforamen or extraforaminal. The most frequent location of lumbar disc herniations are levels L4-5 and L5-S1, whereas the upper lumbar levels are far less commonly affected [4]. It has been shown that image-guided therapeutic injections with steroids are a viable, safe, and effective treatment in patients with lumbar radiculopathy [5–9]. Hence, it is not surprising that the number of these injections has increased over the last few decades, and most likely will continue to do so in the future [10–13]. Lumbar epidural injections can be performed using different approaches: transforaminal epidural steroid injection (TFESI), interlaminar epidural injection, or caudal injection [14]. In case of lumbar radiculopathy, the transforaminal approach is the most widely used; the advantage of TFESI is being more target specific, fulfilling the aim of reaching the ventrolateral epidural space as primary site of pathology [14]. Usually, TFESI is performed by fluoroscopy or computed tomography (CT)-guidance; both modalities are safe and the choice of image modality for needle guidance seems to have no impact on the patient’s outcome [15]. Commonly used agents during TFESI are a combination of a local anesthetic (e.g., lidocaine) and a corticosteroid (e.g., triamcinolone acetate and dexamethasone dihydrogen phosphate). The rationale behind the use of a corticosteroid agent is its long-acting anti-inflammatory effect; radicular pain is commonly caused by mechanical compression of nerves with subsequent local inflammatory cascades, which can thus be inhibited by the local application of the steroid agent and potentially relieve patients’ symptoms related to the radiculopathy for several weeks or months [16, 17]. The application of a local anesthetic during lumbar TFESI is based on the immediate pain reduction effect which may quickly alleviate the pain caused by the nerve compression, and additionally decreases the discomfort caused by the procedure itself [17].

In our institution, we ask each patient before and 15 min after an injection treatment to state their pain level. It would be interesting and beneficial for the patient to know if based on that immediate pain relief, a prediction can be made regarding the longer-term effect of the TFESI. We hypothesize that a correlation exists between the pain score immediately after and the pain score several weeks after the injection. To our knowledge, this potential association has not been investigated thus far.

Therefore, the goal of this study was to examine the correlation between the immediate pain relief after CT-guided TFESI and the longer-term pain relief (4 weeks after TFESI) in patients with single level discogenic lumbar radiculopathy.

## Materials and methods

This prospective single-center study with retrospective data analysis was approved by the local ethics committee. Written informed consent was given by all included subjects prior to the intervention, both for the injection itself as well as for the use of data for research purposes.

### Patient cohort

We used our hospital information system to perform a database search for patients with lumbar radiculopathy who received subsequent CT-guided TFESI in our department.

**Fig. 1:**
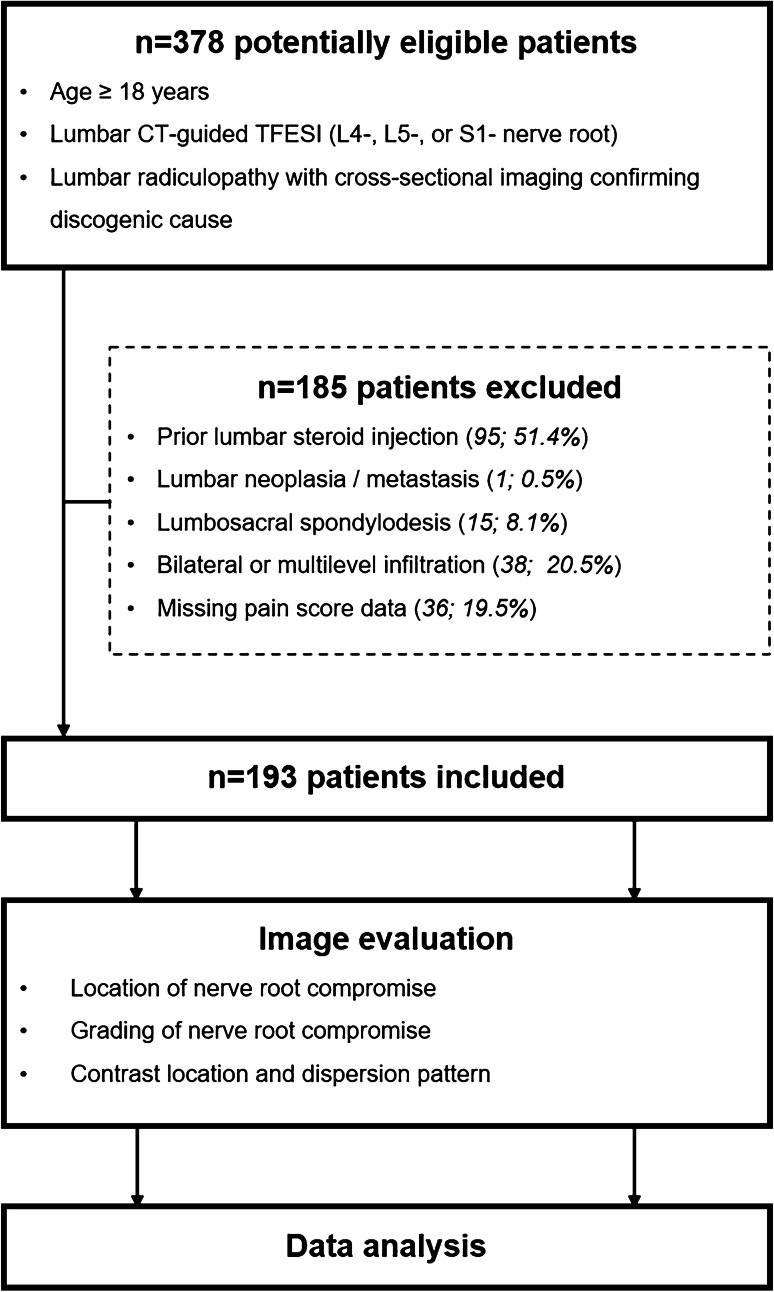
Flowchart of study design. *TFESI*, transforaminal epidural steroid injection

The detailed flow-chart for study inclusion/exclusion is shown in Fig. 1.

Our inclusion criteria were as follows: (1) age ≥ 18 years; (2) CT-guided TFESI for lumbar radiculopathy (L4-, L5- or S1-nerve root), confirmed by clinical examination by either board-certified orthopedic surgeons or rheumatologists; and (3) discogenic nerve compression of the treated level confirmed by either magnetic resonance imaging or CT-myelography within 6 months prior to the injection procedure by a board-certified musculoskeletal radiologist with 8 years of experience.

Exclusion criteria comprised (1) prior lumbar steroid injection; (2) lumbar neoplasia/metastasis; (3) lumbosacral spondylodesis; (4) bilateral or multi-level infiltration during the same session; and (5) missing pain score data.

### Transforaminal epidural steroid injection: technique

**Fig. 2:**
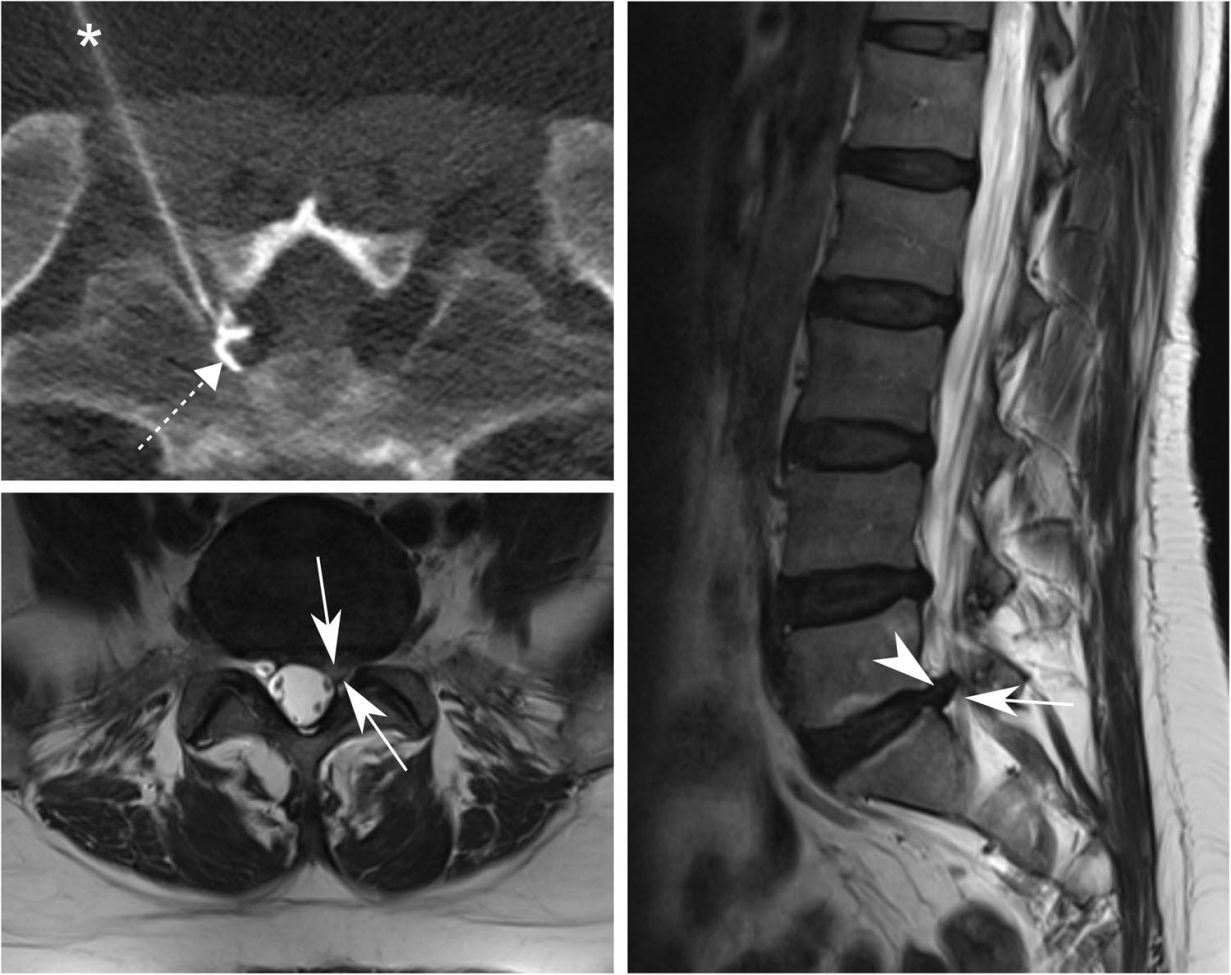
CT-guided TFESI for S1 radiculopathy. A 45-year-old woman with left-sided S1 radiculopathy due to disc herniation (arrowhead) at the L5-S1 level with concomitant grade 2 lateral recess stenosis of the left S1 nerve root (arrows) depicted by transverse (bottom left) and sagittal (right) T2-weighted MR images. CT-guided TFESI (top left) of the S1 nerve root left is shown with correct needle (asterisk) position with the tip adjacent to the nerve root and contrast dispersion (dashed arrow) around the nerve root. *TFESI*, transforaminal epidural steroid injection

**Fig. 3:**
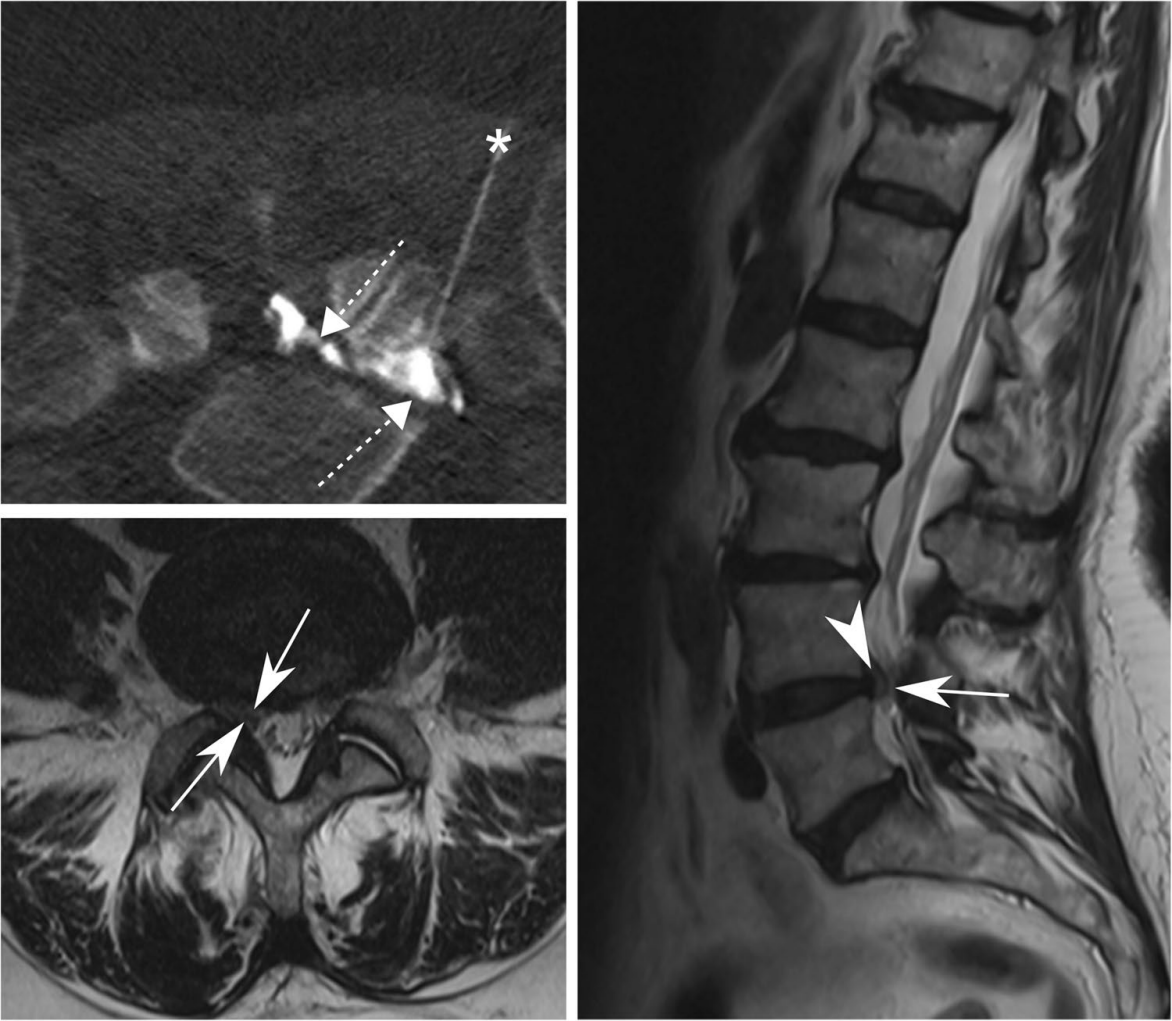
CT-guided TFESI for L5 radiculopathy. A 71-year-old woman with right-sided L5 radiculopathy due to disc herniation (arrowhead) at the L4-5 level with concomitant grade 2 lateral recess stenosis of the right L5 nerve root (arrows), illustrated in transverse (bottom left) and sagittal (right) T2-weighted MR images. CT-guided TFESI (top left) of the right-sided L5 nerve root is depicted with correct needle (asterisk) tip position and contrast dispersion (dashed arrow) reaching the epidural space medially to the lateral recess of the nerve root. *TFESI*, transforaminal epidural steroid injection

All injections were conducted as an outpatient treatment. Each procedure was performed by a fellowship-trained musculoskeletal radiologist. To ensure consistency and reproducibility, a standardized injection protocol was used [7]: (1) initial lumbar low-dose CT in a prone position at the affected level, using a 64-detector row CT; (2) planning the access route for needle insertion; (3) aseptic preparation; (4) needle placement under CT-guidance with the needle tip adjacent to the respective nerve using a transforaminal approach; (5) assure correct needle tip position using iodized contrast agent, 1 mL iopamidol (Iopamiro 200, 200 mg/mL of iodine); (6) injection of 40 mg (1 mL) of triamcinolone acetonide; and (7) injection of 1 mL of 0.2% lidocaine. Figures 2 and 3 illustrate examples of CT-fluoroscopy images during TFESI.

### Image evaluation

**Fig. 4:**
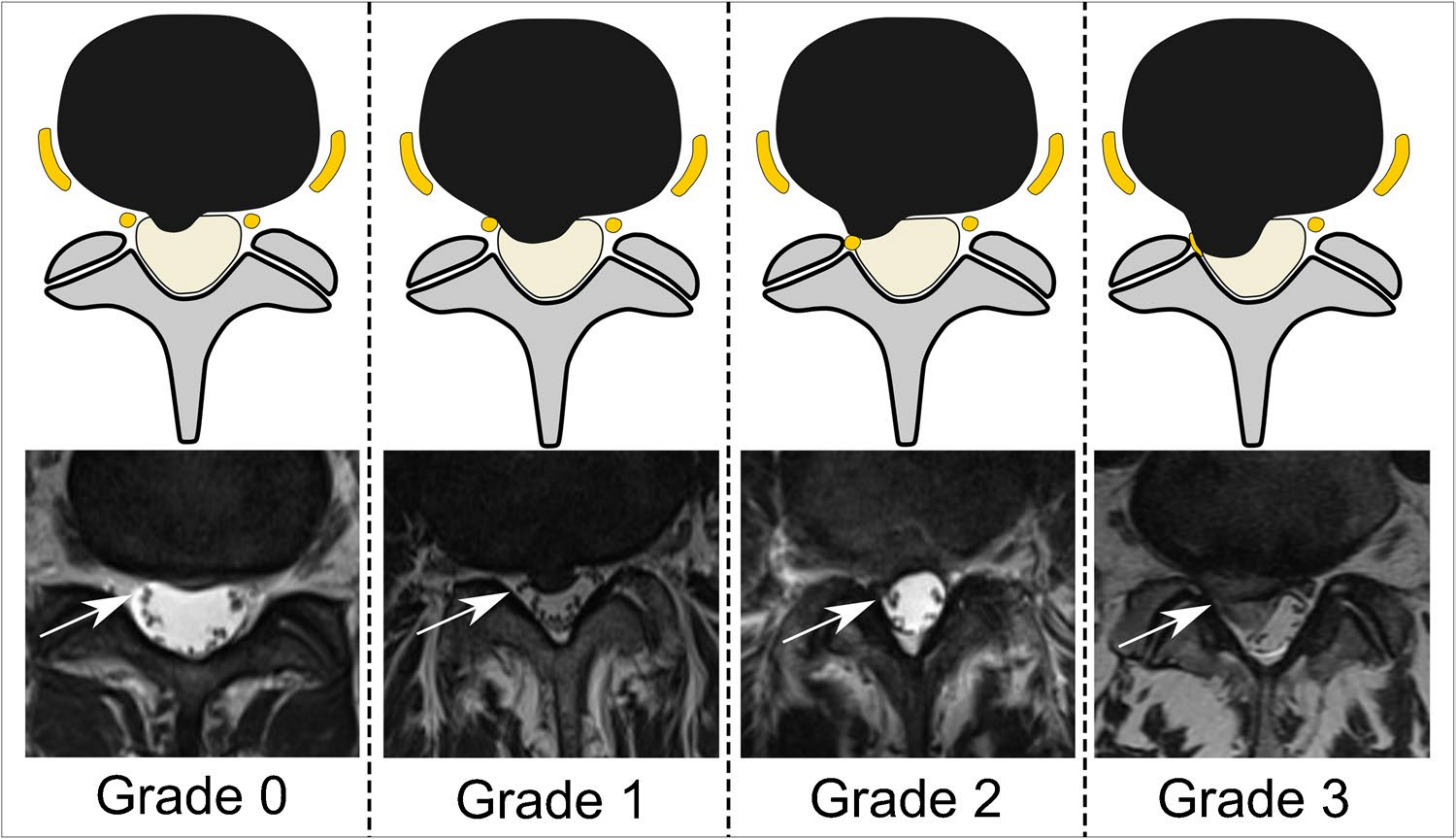
Grading of nerve compression in the lateral recess. Schematic illustration (upper row) and corresponding transverse T2-weighted MR images at the level of the lateral recess (lower row), depicting the grade of nerve root (arrows) compression. Grade 0, “normal/no contact”; Grade 1, “contact”: visible contact of disc material with the nerve root; Grade 2, “deviation”: nerve root is displaced dorsally by disc material; Grade 3, “compression”: nerve root is compressed between disc material and wall of the spinal canal ( adapted from Pfirrmann et. al.) [18]

**Fig. 5:**
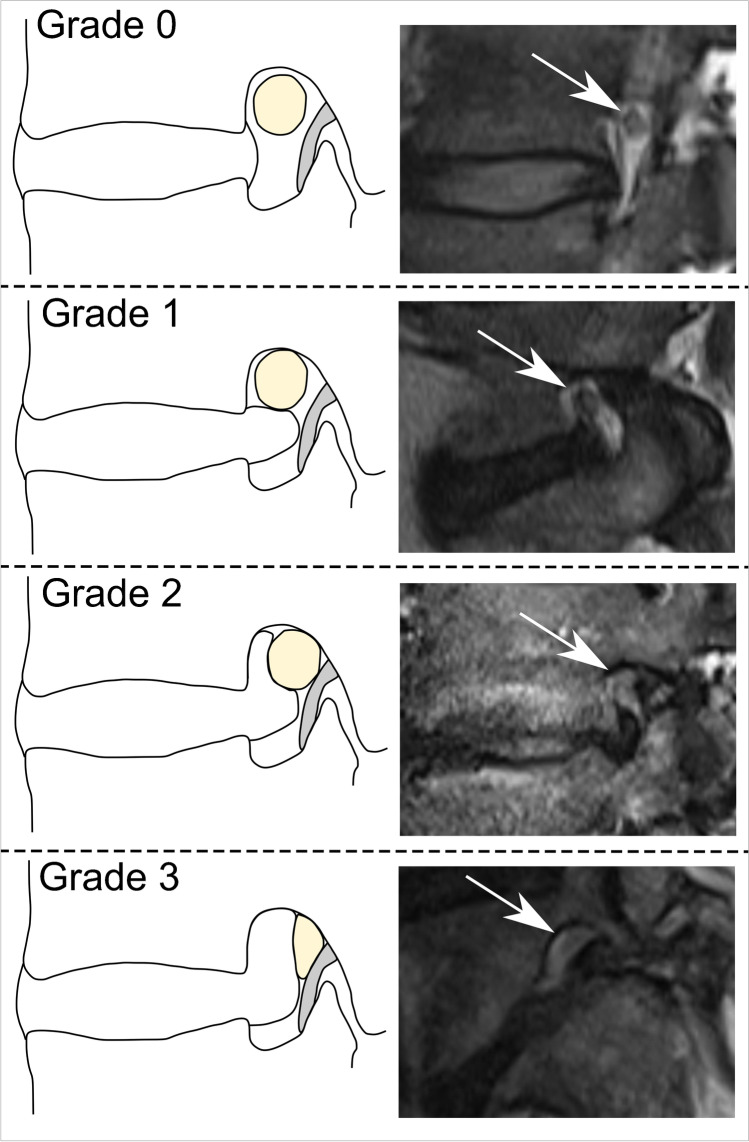
Grading of nerve compression in the neuroforamen. Schematic illustration (left column) and equivalent sagittal T2-weighted MR images at the level of the neuroforamen (right column), depicting the grade of nerve root (arrows) compression. Grade 0, “normal”: perineural fat preserved; Grade 1, “mild stenosis”: perineural fat obliteration in transverse or vertical direction; Grade 2, “moderate stenosis”: perineural fat obliteration in four directions without morphologic change; Grade 3, “severe stenosis”: nerve root collapse/morphologic change ( adapted from Lee et al.) [19]

Cross-sectional pre-procedural imaging studies of the lumbar spine were analyzed on state-of-the-art picture archiving and communication system (PACS) workstations. Image analysis was performed independently by two board-certified fellowship-trained musculoskeletal subspecialized radiologists (*R.S. with 16 years of experience*, *and C.G. with 8 years of experience in musculoskeletal imaging*) blinded to all clinical data. The images were evaluated regarding location of discogenic nerve root compromise of the targeted nerve root (lateral recess, neuroforamen, or extraforaminal); and grading of nerve root compression: grade 0 = no compromise/stenosis; grade 1 = contact of disc with nerve root/mild stenosis, grade 2 = deviation of nerve root/moderate stenosis, and grade 3 = compression of nerve root/severe stenosis [18, 19] (Figs. 4 and 5). Additionally, contrast location and dispersion pattern during the CT-guided TFESI was graded by one radiologist (*C.G. with 8 years of experience in musculoskeletal imaging*) analyzing CT-fluoroscopy images of the lumbar steroid injection. According to the description by Germann et al., the contrast location during CT-TFESI was either extraforaminal, foraminal, or recessal, whereas dispersion pattern was classified as either focal non-linear, linear, or tram-track pattern along the targeted nerve root [7].

### Outcome questionnaire

The outcome evaluation has been performed prospectively using a specifically designed questionnaire. Immediately prior to the TFESI, each patient stated the maximum pain level regarding the low back pain and/or radiating leg pain, using an 11-point numerical rating scale (NRS), serving as baseline reference (NRS_0_): score 0 signifies “no pain” whereas score 10 translates to “intolerable pain.” The same score was evaluated 15 min after the TFESI (NRS_15min_). The pain score 4 weeks after the procedure (NRS_4w_) was acquired using the same questionnaire which was returned to our department via prepaid post. The short-term percental (relative) pain reduction (NRS_%-15 min_) was calculated by subtracting the “NRS_15min_” score from the baseline “NRS_0_” score divided by the “NRS_0_.” Accordingly, the longer-term percental pain reduction (NRS_%-4w_) was calculated by subtracting the “NRS_4w_” score from the baseline “NRS_0_” score divided by the “NRS_0_” score. In order to calculate the predictive performance with odds ratios (OR) of short-term pain reduction on longer-term pain reduction, two groups were formed based on the pain score reduction 4 weeks after TFESI in relation to the preprocedural pain score (NRS_0_): (1) “good responder” with at least 50% reduction in NRS score and (2) “poor responder” with below 50% reduction in NRS score 4 weeks after the peri-radicular injection [7, 8, 20].

In addition to the NRS pain score, the patient global impression of change (PGIC, a seven-item scale), as an item of measurement of the patient’s quality of life, was assessed 4 weeks after the injection; each participant was asked to rate the overall change in activity limitation, symptoms, emotions, and overall quality of life related to the low back pain and radiating leg pain after the TFESI [7, 15, 21, 22]. The possible answers included (1) “much worse,” (2) “worse,” (3) slightly worse,” (4) “no change,” (5) “slightly better,” (6) “better,” and (7) “much better.”

### Statistical analysis

Statistical analysis was performed using SPSS (v25, IBM Corp., Somers, NY). General descriptive statistics were applied. Continuous data are presented as means with standard deviation and categorical data are presented as percentages. Inter-reader reliability was assessed using Cohen’s κ for each qualitative variable and interpreted according to Kundel and Polansky [23] as either “slight” (0–0.20), “fair” (0.21–0.40), “moderate” (0.41–0.60), “substantial” (0.61–0.80), or “almost perfect” agreement (0.81–1.00). Spearman’s rank correlation was used to test for association between two continuous variables or between a continuous and ordinal variable. Correlation analysis between dichotomous (binomial) variables was achieved by means of point-biserial correlation. The strength of an association was expressed as odds ratio (*OR*) with 95% confidence intervals (*CI*). A *p*-value < 0.05 was considered to represent statistical significance.

## Results

### Inter-observer agreement

The agreement between reader 1 and 2 was almost perfect for assessing the grade of “foraminal stenosis” (*κ* = 0.83) and perfect for evaluating the presence of “extraforaminal stenosis” (*κ* = 1.0). The agreement was substantial for assessing the grade of “recessal stenosis” (*κ* = 0.70).

### Demographics

After applying the inclusion and exclusion criteria (Fig. 1), 193 patients were enrolled in this study. Patient characteristics are shown in Table1.

**Table 1:** Patient characteristics. Demographic variables gender, age, side, level, and pain assessment are shown. The NRS scores are given for three time points: (1) before the TFESI (NRS_0_), (2) 15 min after the TFESI (NRS_15min_), and (3) 4 weeks after the TFESI (NRS_4w_). NRS_%-15 min_ corresponds to the relative pain score reduction 15 min after the TFESI, and NRS_%-4w_ represents pain score reduction 4 weeks after the TFESI. Qualitative variables are given in numbers (percentages), and continuous variables are presented as mean ± standard deviation. *NRS*, numerical rating scale

**Variable**	
Gender, *n* (%)	
Male	92 (47.7)
Female	101 (52.3)
Age, years	55.4 ± 14.9
Side, *n* (%)	
Left	110 (57.0)
Right	83 (43.0)
Level, *n* (%)	
L4	29 (15.0)
L5	88 (45.6)
S1	76 (39.4)
Pain assessment	
NRS_0_/NRS_15min_/NRS_4w_	6.1 ± 2.0/3.6 ± 2.1/3.6 ± 2.5
NRS_%-15 min_/NRS_%-4w_	38.3% ± 33.6%/37.6% ± 47.9%

### Treatment outcome: NRS and PGIC score

NRS scores are given in Table 1. The pain score was 6.1 ± 2.0 before the TFESI (NRS_0_), 3.6 ± 2.1 15 min after the injection (NRS_15min_), and 3.6 ± 2.5 4 weeks after the procedure (NRS_4w_). This translates to a short-term percental pain score reduction (NRS_%-15 min_) of 38.3% ± 33.6% and a longer-term percental pain score reduction (NRS_%-4w_) of 37.6% ± 47.9%.

The PGIC score 4 weeks after the TFESI (available for *n* = 189 patients) was as follows: “much worse” in 43 of 189 patients (22.8%), “worse” in 42 of 189 patients (22.2%), “slightly worse” in 50 of 189 patients (26.5%), “no change” in 32 of 189 patients (16.9%), “slightly better” in 7 of 189 patients (3.7%), “better” in 11 of 189 patients (5.8%), and “much better” in 4 of 189 patients (2.1%), respectively.

### Imaging findings

The frequency of imaging findings regarding nerve root compromise (location and grade) and contrast location/dispersion pattern during CT-guided TFESI are shown in Table 2.

**Table 2:** Imaging findings. The presence and grade of nerve root compromise are given for the locations extraforaminal, foraminal, and lateral recess. Additionally, contrast location and contrast dispersion pattern during CT-guided TFESI are given for Reader 2. Data are presented as numbers (percentages). *Contrast location and contrast dispersion pattern was analyzed for *n* = 172 subjects, as contrast medium was not given or respective CT-fluoroscopy images were not available in 21 subjects. *TFESI*, transforaminal epidural steroid injection

Variable	*Reader 1*	*Reader 2*
*Extraforaminal stenosis*,* n* (%)		
Yes	4 (2.1)	4 (2.1)
No	189 (97.9)	189 (97.9)
*Foraminal stenosis*, *n* (%)		
Grade 0	99 (51.3)	113 (58.5)
Grade 1	46 (23.8)	35 (18.1)
Grade 2	14 (7.3)	16 (8.3)
Grade 3	34 (17.6)	29 (15.0)
*Lateral recess stenosis*,* n* (%)		
Grade 0	11 (5.7)	15 (7.8)
Grade 1	27 (14.0)	34 (17.6)
Grade 2	57 (29.5)	60 (31.1)
Grade 3	98 (50.8)	84 (43.5)
Contrast location*		
Extraforaminal	-	56 (32.5)
Foraminal	-	82 (47.7)
Lateral recess	-	34 (19.8)
Contrast dispersion pattern*		
Focal non-linear	-	39 (22.7)
Linear	-	116 (67.4)
Tram-track	-	17 (9.9)

### Correlation analysis between outcome measures after TFESI

Detailed data of each correlation analysis are presented in Table 3. The baseline NRS score “NRS_0_” showed a significant positive correlation with the NRS score after 15 min “NRS_15min_” (*r* = 0.29; *p* < 0.001) and with the NRS score after 4 weeks “NRS_4w_” (*r* = 0.22; *p* = 0.002).

**Table 3:** Correlation analysis: NRS and PGIC. Data represents correlation analysis between the various outcome parameters and time points after TFESI. NRS_0_, pain score prior to TFESI; NRS_15min_, pain score 15 min after TFESI; NRS_4w_, pain score 4 weeks after TFESI; NRS_%-15 min_, relative/percental pain score reduction 15 min after TFESI; NRS_%-4w_, relative/percental pain score reduction 4 weeks after TFESI; PGIC_4w_, patient global impression of change 4 weeks after TFESI. *Denotes statistical significance (*p* < 0.05). *NRS*, numerical rating scale. *TFESI*, transforaminal epidural steroid injection

Correlation analysis of outcome measures after TFESI		
**Variable pair**	***Correlation coefficient***	***p-value***
*NRS*_0_ *│ NRS*_15min_	**0.29**	< 0.001*
*NRS*_0_ *│ NRS*_4w_	**0.22**	0.002*
*NRS*_0_ *│* NRS_%-15 min_	**0.22**	0.002*
*NRS*_0_ *│* NRS_%-4w_	**0.19**	0.009*
*NRS*_15min_ *│ NRS*_4w_	**0.25**	< 0.001*
*NRS*_%-15 min_ *│* NRS_%-4w_	**0.24**	0.001*
*NRS*_%-15 min_ *│* PGIC_4w_	− 0.10	0.18

The “NRS_0_” revealed a significant positive association both with the relative pain reduction after 15 min “NRS_%-15 min_” (*r* = 0.22; *p* = 0.002) and with the pain relief after 4 weeks (*r* = 0.19; *p* = 0.009).

Our results further indicate a significant positive correlation between the short-term NRS score after the procedure “NRS_15min_” and the longer-term NRS score “NRS_4w_” (*r* = 0.25; *p* < 0.001).

The short-term pain reduction after 15 min “NRS_%-15 min_” was significantly and positively associated with the longer-term pain relief “NRS_%-4w_” (*r* = 0.24; *p* = 0.001).

No significant correlation occurred between the pain reduction 15 min after the TFESI “NRS_%-15 min_” and the PGIC score 4 weeks after injection “PGIC_4w_” (*p* = 0.18).

### Predictive value of short-term pain relief

The cross-table between short-term and longer-term pain relief with odds ratio calculation is shown in Table 4. The short-term treatment response was a correct predictor for longer-term pain reduction (either persistent good response or persistent poor response) in 113 of 193 patients (67.4%). A good response 15 min after TFESI (NRS_%-15 min_” ≥ 50%) led to a good longer-term response after 4 weeks (NRS_%-4w_” ≥ 50%) in 59.7% (95% *CI*: 50.9 to 68.0%) of patients. An unfavorable pain relief 15 min after TFESI (NRS_%-15 min_” < 50%) continued to be a poor longer-term pain relief (NRS_%-4w_” < 50%) in 57.8% (95% *CI*: 51.9 to 63.4%) of subjects.

**Table 4:** Odds ratio for ≥ 50% pain reduction after 4 weeks. NRS_%-15 min_, relative/percental pain score reduction 15 min after TFESI; NRS_%-4w_, relative/percental pain score reduction 4 weeks after TFESI. *Denotes statistical significance (*p* < 0.05). ^**#**^*p*-value derived from logistic regression. *CI*, confidence interval; *NRS*, numerical rating scale; *OR*, odds ratio

Odds ratio for ≥ 50% pain reduction after 4 weeks depending on immediate pain relief							
		**NRS**_**%-4w**_** ≥ 50%**			*OR*	95% *CI*	*p*-value^**#**^
		**Yes**		**No**			
**NRS**_**%-15 min**_** ≥ 50%**	**No**	42.2%	57.8%		1.0		
	**Yes**	59.7%	40.3%		2.0	1.1–3.6	0.018*

Presuming a good short-term pain reduction (NRS_%-15 min_” ≥ 50%), the odds ratio of a good longer-term pain relief (NRS_%-4w_” ≥ 50%) was 2.0 (95% *CI*: 1.1 to 3.6). This means patients with a good longer-term outcome (≥ 50% pain score reduction) 4 weeks after TFESI are twice as likely to have already shown a good short-term outcome (≥ 50%) immediately after the injection.

### Influence of demographic variables on pain relief

Correlation analysis between demographics and pain relief is depicted in Table 5.

**Table 5:** Correlation analysis: demographic variables and pain relief. Data represents correlation analysis between demographic variables and pain relief 15 min and 4 weeks after TFESI. Numbers represent correlation coefficients (*p*-values). Spearman’s rank correlation (°) or point-biserial correlation (#) was applied. NRS_%-15 min_, relative/percental pain score reduction 15 min after TFESI; NRS_%-4w_, relative/percental pain score reduction 4 weeks after TFESI. *Denotes statistical significance (*p* < 0.05). *NRS*, numerical rating scale; *TFESI*, transforaminal epidural steroid injection

Correlation analysis between patient characteristics and pain relief after TFESI		
**Variable**	**NRS**_**%-15 min**_ Correlation coefficient (*p*-value)	**NRS**_**%-4w**_ Correlation coefficient (*p*-value)
^*#*^*Gender*	0.03 (0.72)	0.01 (0.89)
^*°*^*Age*	0.16 (0.02)*	0.09 (0.23)
^*#*^*Side*	0.04 (0.54)	− 0.005 (0.95)
^*°*^*Level*	− 0.09 (0.24)	− 0.05 (0.49)

There was a weak but significant positive correlation between age and short-term pain reduction “NRS_%-15 min_” (*r* = 0.16; *p* = 0.02; “the older the patient, the better the pain reduction”), but no significant association between age and long-term pain reduction “NRS_%-4w_” (*p* = 0.23).

Fifteen minutes after the TFESI, gender (*p* = 0.72), side of TFESI (*p* = 0.54), and level of TFESI (*p* = 0.24) were not associated with the pain reduction “NRS_%-15 min_.”

Four weeks after the TFESI, neither gender (*p* = 0.89), nor side of treatment (*p* = 0.95), nor level of steroid injection (*p* = 0.49) correlated with the pain reduction “NRS_%-4w_.”

### Influence of imaging findings on pain relief

Data of correlation analysis between imaging findings and pain relief are illustrated in Table 6.

**Table 6:** Correlation analysis: imaging findings and pain relief. Data represents correlation analysis between imaging findings and pain relief 15 min and 4 weeks after TFESI. Numbers represent correlation coefficients (*p*-values). Spearman’s rank correlation (°) or point-biserial correlation (#) was applied. NRS_%-15 min_, relative/percental pain score reduction 15 min after TFESI; NRS_%-4w_, relative/percental pain score reduction 4 weeks after TFESI. *Denotes statistical significance (*p* < 0.05). *NRS*, numerical rating scale; *TFESI*, transforaminal epidural steroid injection

Correlation analysis between imaging findings and pain relief after TFESI		
Variable	NRS_%-15min_ Correlation coefficient (*p*-value)	NRS_%-4w_ Correlation coefficient (*p*-value)
^*#*^*Extraforaminal stenosis (Binary: yes or no)*		
Reader 1	0.001 (0.99)	< 0.001 (1.0)
Reader 2	0.001 (0.99)	< 0.001 (1.0)
^*°*^*Foraminal stenosis (Grade 0–3)*		
Reader 1	0.01 (0.87)	0.01 (0.87)
Reader 2	−0.03 (0.69)	−0.01 (0.86)
^*°*^*Lateral recess stenosis* *(Grade 0–3)*		
Reader 1	−0.15 (0.03)*	0.11 (0.12)
Reader 2	0.10 (0.19)	0.13 (0.07)
^*#*^*Location of stenosis (Binary: foraminal or recessal)*		
Reader 1	0.03 (0.76)	0.11 (0.26)
Reader 2	0.07 (0.42)	0.14 (0.12)
^*°*^*Contrast location*		
Reader 2	−0.09 (0.22)	−0.05 (0.48)
^*°*^*Contrast dispersion pattern*		
Reader 2	−0.12 (0.11)	0.03 (0.74)

A weak but significant positive correlation was seen between the grading of nerve root compromise at the lateral recess and short-term pain relief “NRS_%-15 min_” (“the lower the grade, the better the pain relief”) only for reader 1 (*r* =  − 0.15, *p* = 0.03), whereas no significant association was found between grading of nerve root compromise at the lateral recess and longer-term pain relief “NRS_%-4w_” for both reader (reader 1: *p* = 0.12; reader 2: *p* = 0.07). No correlation was found between presence of extraforaminal stenosis and pain relief (all *p* ≥ 0.99), between grading of foraminal stenosis and pain relief (all *p* ≥ 0.69), and between location of stenosis and pain relief (all *p* ≥ 0.12) both at 15 min and 4 weeks after the injection. Furthermore, neither the contrast location nor the contrast dispersion pattern during TFESI had an influence on short-term pain relief (*p* = 0.22 and *p* = 0.11) or longer-term pain relief (*p* = 0.48 and *p* = 0.74).

### Complications

No minor or major complication after CT-guided TFESI was reported.

## Discussion

Lumbar transforaminal epidural steroid injection (TFESI) is a very common procedure with good outcomes [5–8]. So far however, we were not able to tell patients after lumbar TFESI how high the likelihood of a persisting good pain reduction after 4 weeks is if the immediate pain relief after the injection was already good. In this prospective study with retrospective data analysis, we investigated the relationship between (1) short-term pain relief (effect of local anesthetics) and (2a) longer-term pain reduction (effect of steroids) or (2b) patient global impression of change (PGIC) after CT-guided lumbar TFESI in patients with unilateral single-level discogenic radiculopathy.

Our results show the efficacy of CT-guided TFESI; the mean reduction of the NRS pain score was 38.3% ± 33.6% after 15 min and 37.6% ± 47.9% after 4 weeks, which is within the range of findings in comparable studies [5, 7, 24–26].

Our results further indicate a significant positive correlation between the immediate pain score reduction 15 min after TFESI and the longer-term pain relief 4 weeks later, with an odds ratio of 2.0. This signifies that patients with a good longer-term outcome (≥ 50% pain score reduction) 4 weeks after TFESI are twice as likely to have already shown a good short-term outcome (≥ 50%) immediately after the injection. Nearly 60% of patients with a good short-term response (≥ 50% pain score reduction) continue to maintain a longer-term good response after 4 weeks. The short-term treatment response was a correct predictor for longer-term pain reduction (either persistent good response or persistent poor response) in 113 of 193 patients (67.4%). However, approximately 40% of patients with an initially positive treatment response (≥ 50% pain score reduction after 15 min) will develop an unfavorable pain score reduction (< 50%) after 4 weeks. One reason for this discrepancy may be attributed to the different pharmacological effect of local anesthetics (which reflect the short-term outcome) compared to steroids (which reflect the longer-term outcome); local anesthetic agents interact — among others — with specific ionic channels and receptors along the targeted nerve, eventually interrupting afferent pain impulses [27]. However, steroids are used in TFESI because of their inhibiting effect on local inflammatory cascades, which are generated by mechanical irritation of the nerve, for example caused by a disc herniation [16].

To our knowledge, our study is the first to investigate the association between immediate pain reduction and longer-term pain relief after lumbar TFESI. For the cervical spine, Antoniadis et al. and Wald et al. examined the predictive value of post-procedural pain relief for longer-term pain reduction after CT-guided cervical nerve root injection with local anesthetics and steroids [28, 29]. In contrast to our findings at the lumbar spine, both those studies observed no correlation between immediate pain relief after injection and longer-term pain relief (6–8 weeks later) in patients with cervical radiculopathy. This discrepancy may be explained by the different injection technique (indirect approach to the nerve root for cervical injection versus direct approach for lumbar injection in our study) and/or a different steroid agent (non-particulate for the cervical injection versus particulate steroids for lumbar TFESI in our study). Moreover, a smaller patient cohort in the study by Antoniadis and coworkers (34 for the cervical injection versus 193 for the lumbar TFESI in our study) maybe another reason for the discrepant findings [28].

Furthermore, we encountered a moderate positive correlation between the baseline preprocedural pain score NRS_0_ and both the pain 15 min (NRS_15min_) and 4 weeks (NRS_4w_) after the TFESI, which is consistent with findings by Tagowski et al., although the correlation in that study was seen for the subgroup of patients with lumbar TFESI using a non-particulate steroid, as opposed to particulate steroids in our study [8].

The short-term pain relief after 15 min did not correlate with the patients’ global impression of change (PGIC) 4 weeks after the treatment. This nonexistent association may be explained by the composition of the PGIC score, which reflects not only pain but also activity limitation, symptoms, emotions, and overall quality of life [21].

No confounding patient characteristics (gender, age, side of treatment, and level of treated radiculopathy) were detected regarding longer-term pain reduction 4 weeks after TFESI. Patient age was the only demographic variable that was significantly and positively correlated with short-term pain reduction after 15 min; however, the association was weak and arguably negligible.

Regarding pre-procedural imaging findings, the nerve root compression grade at the lateral recess correlated significantly with the short-term pain reduction 15 min after TFESI for only one reader. Nevertheless, the association was weak and therefore clinically presumably irrelevant, in particular because this positive correlation did not persist for lateral recess nerve compromise grading and longer-term pain relief after 4 weeks. The presence and grading of foraminal and/or extraforaminal nerve compression, as well as the location of nerve compression (foramen versus lateral recess) was not associated with pain score reduction at both time points, which is consistent with findings in other studies [8, 30]. Furthermore, neither the contrast location nor the contrast dispersion pattern along the targeted nerve root during CT-guided TFESI had an influence on pain relief after 15 min or 4 weeks, confirming results of a recent study [7].

Our study has limitations. First, we acknowledge that the exact time of longer-term outcome assessment after injection might vary within our cohort, as each patient reported the treatment response on a questionnaire via prepaid post which was handed out at the date of the injection. Therefore, presumably not each patient completed the questionnaire exactly 4 weeks after the procedure, which may potentially bias the results. Second, our follow-up period ended after 4 weeks and does not reflect the true long-term effect (several months to years), which might differ from the presented results. Due to the retrospective nature of this study, no outcome data was available for this time period. Third, a certain variability in the TFESI procedure is possible when performed by different radiologists. However, it is a single-center study and each radiologist received a strict fellowship training using highly standardized interventional protocols. Additionally, the needle tip position directly adjacent to the treated nerve during the CT-guided TFESI was confirmed in each included case, providing a homogenous cohort regarding injection procedure. Furthermore, we acknowledge that when located within the neuroforamen, the discogenic nerve compression is sometimes accompanied by either osteoarthrosis of the facet joint and/or hypertrophy of the ligamentum flavum instead of being purely discogenic, which might influence the treatment response. Despite these limitations, the considerable cohort size and application of strict inclusion and exclusion criteria establish a firm basis for the reliability of our findings.

In conclusion, our study demonstrates a positive correlation between immediate post-procedural pain reduction after CT-guided TFESI (effect of local anesthetic) and longer-term pain relief (effect of steroid) in patients with single-level discogenic lumbar radiculopathy. Patients with a good outcome (≥ 50% pain score reduction) 4 weeks after TFESI are 2 times more likely to have already shown a good short-term outcome (≥ 50%) immediately after the injection. Additionally, short-term pain relief is no predictor for patients’ global impression of change 4 weeks after TFESI.

## Abbreviations

CI: Confidence interval
NRS: Numerical rating scale
PGIC: Patient global impression of change
TFESI: Transforaminal epidural steroid injection

## References

[1] Wenger HC, Cifu AS (2017). Treatment of low back pain. JAMA.

[2] Hoy D, Brooks P, Blyth F, Buchbinder R (2010). The epidemiology of low back pain. Best Pract Res Clin Rheumatol.

[3] Manchikanti L, Singh V, Datta S, Cohen SP, Hirsch JA, American Society of Interventional Pain P. Comprehensive review of epidemiology, scope, and impact of spinal pain. Pain Physician. 2009; 12(4):E35–70. 19668291

[4] Tarulli AW, Raynor EM (2007). Lumbosacral radiculopathy. Neurol Clin.

[5] Bensler S, Sutter R, Pfirrmann CWA, Peterson CK (2018). Particulate versus non-particulate corticosteroids for transforaminal nerve root blocks: Comparison of outcomes in 494 patients with lumbar radiculopathy. Eur Radiol.

[6] Peterson CK, Humphreys BK, Hodler J, Pfirrmann CW (2012). Gender differences in pain levels before and after treatment: a prospective outcomes study on 3,900 Swiss patients with musculoskeletal complaints. BMC Musculoskelet Disord.

[7] Germann C, Graf DN, Fritz B, Sutter R. CT-guided transforaminal epidural steroid injection for discogenic lumbar radiculopathy: influence of contrast dispersion and radiologist’s experience on clinical outcome. Skeletal Radiology. 2021. 10.1007/s00256-021-03881-x PMC8854304 34382098

[8] Tagowski M, Lewandowski Z, Hodler J, Spiegel T, Goerres GW (2019). Pain reduction after lumbar epidural injections using particulate versus non-particulate steroids: intensity of the baseline pain matters. Eur Radiol.

[9] Manchikanti L, Knezevic E, Knezevic NN, Sanapati MR, Thota S, Abd-Elsayed A (2021). Epidural injections for lumbar radiculopathy or sciatica: a comparative systematic review and meta-analysis of cochrane review. Pain Physician.

[10] Manchikanti L, Pampati V, Boswell MV, Smith HS, Hirsch JA (2010). Analysis of the growth of epidural injections and costs in the Medicare population: a comparative evaluation of 1997, 2002, and 2006 data. Pain Physician.

[11] Manchikanti L, Pampati V, Falco FJ, Hirsch JA (2013). Assessment of the growth of epidural injections in the medicare population from 2000 to 2011. Pain Physician.

[12] Manchikanti L, Pampati V, Falco FJ, Hirsch JA (2015). An updated assessment of utilization of interventional pain management techniques in the Medicare population: 2000–2013. Pain Physician.

[13] Manchikanti L, Sanapati MR, Soin A, Manchikanti MV, Pampati V, Singh V (2020). An updated analysis of utilization of epidural procedures in managing chronic pain in the Medicare population from 2000 to 2018. Pain Physician.

[14] Buenaventura RM, Datta S, Abdi S, Smith HS (2009). Systematic review of therapeutic lumbar transforaminal epidural steroid injections. Pain Physician.

[15] Dietrich TJ, Peterson CK, Zeimpekis KG, Bensler S, Sutter R, Pfirrmann CWA (2019). Fluoroscopy-guided versus CT-guided lumbar steroid injections: comparison of radiation exposure and outcomes. Radiology.

[16] Marshall LL, Trethewie ER (1973). Chemical irritation of nerve-root in disc prolapse. Lancet.

[17] Knezevic NN, Manchikanti L, Urits I, Orhurhu V, Vangala BP, Vanaparthy R (2020). Lack of superiority of epidural injections with lidocaine with steroids compared to without steroids in spinal pain: a systematic review and meta-analysis. Pain Physician.

[18] Pfirrmann CW, Dora C, Schmid MR, Zanetti M, Hodler J, Boos N (2004). MR image-based grading of lumbar nerve root compromise due to disk herniation: reliability study with surgical correlation. Radiology.

[19] Lee S, Lee JW, Yeom JS, Kim KJ, Kim HJ, Chung SK (2010). A practical MRI grading system for lumbar foraminal stenosis. AJR Am J Roentgenol.

[20] Jung YS, Suh JH, Kim HY, Min K, Oh Y, Park D (2016). The prognostic value of enhanced-MRI and fluoroscopic factors for predicting the effects of transforaminal steroid injections on lumbosacral radiating pain. Ann Rehabil Med.

[21] Fischer D, Stewart AL, Bloch DA, Lorig K, Laurent D, Holman H (1999). Capturing the patient’s view of change as a clinical outcome measure. JAMA.

[22] Newell D, Bolton JE. Responsiveness of the Bournemouth questionnaire in determining minimal clinically important change in subgroups of low back pain patients. Spine (Phila Pa 1976). 2010; 35(19):1801–1806. 10.1097/BRS.0b013e3181cc006b 20581759

[23] Kundel HL, Polansky M (2003). Measurement of observer agreement. Radiology.

[24] Bensler S, Sutter R, Pfirrmann CWA, Peterson CK (2017). Is there a difference in treatment outcomes between epidural injections with particulate versus non-particulate steroids?. Eur Radiol.

[25] Chang MC, Lee DG (2018). Outcome of transforaminal epidural steroid injection according to the severity of lumbar foraminal spinal stenosis. Pain Physician.

[26] Makkar JK, Gourav KKP, Jain K, Singh PM, Dhatt SS, Sachdeva N (2019). Transforaminal versus lateral parasagittal versus midline interlaminar lumbar epidural steroid injection for management of unilateral radicular lumbar pain: a randomized double-blind trial. Pain Physician.

[27] Lirk P, Hollmann MW, Strichartz G (2018). The science of local anesthesia: basic research, clinical application, and future directions. Anesth Analg.

[28] Antoniadis A, Dietrich TJ, Farshad M (2016). Does pain relief by CT-guided indirect cervical nerve root injection with local anesthetics and steroids predict pain relief after decompression surgery for cervical nerve root compression?. Acta Neurochir.

[29] Wald JT, Maus TP, Geske JR, Diehn FE, Kaufmann TJ, Murthy NS (2013). Immediate pain response does not predict long-term outcome of CT-guided cervical transforaminal epidural steroid injections. AJNR Am J Neuroradiol.

[30] Bensler S, Walde M, Fischer MA, Pfirrmann CW, Peterson CK, Sutter R (2020). Comparison of treatment outcomes in lumbar disc herniation patients treated with epidural steroid injections: interlaminar versus transforaminal approach. Acta Radiol.

